# Transient Idiopathic Isolated Unilateral Hypoglossal Nerve Palsy

**DOI:** 10.1007/s11606-012-2228-9

**Published:** 2012-10-05

**Authors:** Kiyoshi Shikino, Kazutaka Noda, Masatomi Ikusaka

**Affiliations:** Department of General Medicine, Chiba University Hospital, 1-8-1, Inohana, Chuo-ku, Chiba 260-8670 Japan

**Keywords:** hypoglossal nerve palsy, isolated, unilateral, idiopathic, transient

**Figure 1.**
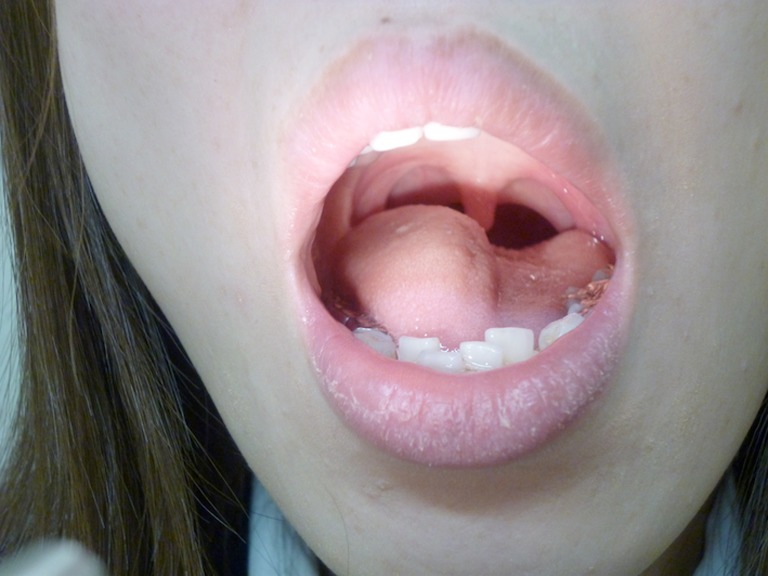
Right tongue mounding at rest.

**Figure 2.**
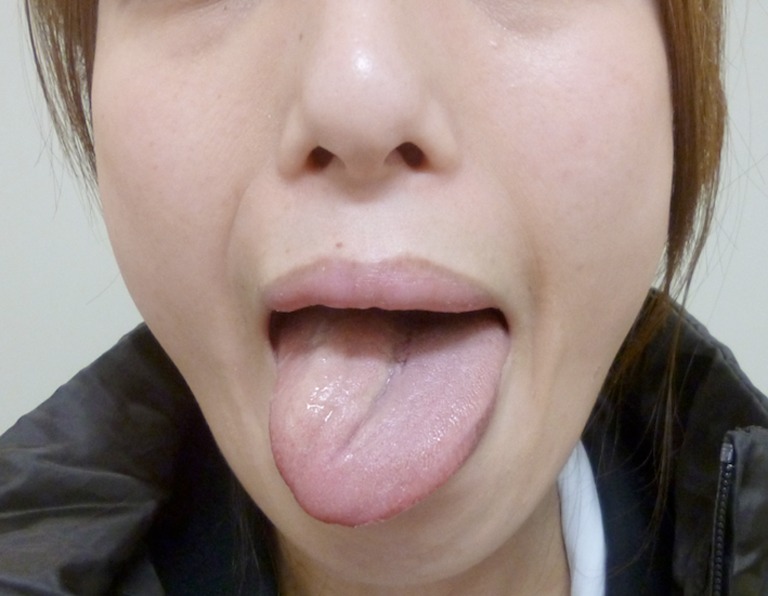
Tongue deviation to the right.

A 33-year-old woman presented with five days of dysarthria and dysphagia. Her past medical history included Graves’ disease treated with a partial thyroidectomy five years earlier. On examination, she had tongue mounding and deviation to the right (Figs. 1 and 2). Other neurological and otorhinolaryngological findings were normal. Titers of herpes simplex virus and varicella-zoster virus antibodies were positive, but did not differ between the acute and convalescent phase sera. Brain magnetic resonance imaging (MRI) and magnetic resonance angiogram (MRA) findings were normal, and laboratory tests showed no evidence of inflammation. Idiopathic isolated unilateral hypoglossal nerve palsy was diagnosed.

While cranial nerves III, IV, VI and VII are more prone to idiopathic palsy, idiopathic unilateral hypoglossal nerve palsy is rare. With the limited number of cases reported, the male-to-female ratio appears even, the age of onset is widely distributed (20–69 years) and the majority of cases self-resolve.^1^ In acute isolated hypoglossal nerve palsy, malignancy, vascular pathology, herpes infection, systemic lupus erythematosus, multiple sclerosis and diabetic mononeuropathy have been reported as causes.^1^–^3^ Although the paired sera failed to show acute herpes infection in this case, reactivation of the disease could still be possible. Eight weeks after symptom onset, this patient fully recovered without intervention.
